# Gastrointestinal Microenvironment and the Gut-Lung Axis in the Immune Responses of Severe COVID-19

**DOI:** 10.3389/fmolb.2021.647508

**Published:** 2021-04-12

**Authors:** Yun Yang, Weishan Huang, Yubo Fan, Guo-Qiang Chen

**Affiliations:** ^1^ Beijing Advanced Innovation Centre for Biomedical Engineering, Key Laboratory for Biomechanics and Mechanobiology of Ministry of Education, School of Biological Science and Medical Engineering, Beihang University, Beijing, China; ^2^ Department of Pathobiological Sciences, School of Veterinary Medicine, Louisiana State University, Baton Rouge, LA, United States; ^3^ Department of Microbiology and Immunology, College of Veterinary Medicine, Cornell University, Ithaca, NY, United States; ^4^ Center for Synthetic and Systems Biology, School of Life Sciences, Tsinghua University, Beijing, China; ^5^ MOE Key Lab of Industrial Biocatalysis, Department of Chemical Engineering, Tsinghua University, Beijing, China; ^6^ Tsinghua-Peking Center for Life Sciences, School of Life Sciences, Tsinghua University, Beijing, China

**Keywords:** severe COVID-19, SARS-CoV-2, gut-lung axis, enteric infection, intestinal dysbiosis

## Abstract

The global pandemic of coronavirus disease 2019 (COVID-19), caused by severe acute respiratory syndrome coronavirus 2 (SARS-CoV-2), is an unprecedented threat to the human health. A close association of the digestive tract is implied by the high frequency of gastrointestinal syndromes among COVID-19 patients. A better understanding of the role of intestinal microenvironment in COVID-19 immunopathology will be helpful to improve the control of COVID-19 associated morbidity and mortality. This review summarizes the immune responses associated with the severity of COVID-19, the current evidence of SARS-CoV-2 intestinal tropism, and the potential involvement of gut microenvironment in COVID-19 severity. Additionally, we discuss the therapeutic potential of probiotics as an alternative medicine to prevent or alleviate severe COVID-19 outcome.

## Introduction

Severe acute respiratory syndrome coronavirus 2 (SARS-CoV-2) is a novel zoonotic coronavirus with human-to-human transmission route, and its recent emergence and rapid spread has resulted in a global pandemic of coronavirus disease 2019 (COVID-19) (Zhou P. et al., 2020). The common clinical symptoms of COVID-19 patients include fever, cough, fatigue and pulmonary pneumonia, however, around 20% of the SARS-CoV-2 infected individuals developed acute respiratory failure and even fatal outcomes (Aggarwal et al., 2020; Chen N. et al., 2020). By February 15, 2021, more than 108,000,000 confirmed cases of infection and 2,390,000 deaths have been reported globally, making SARS-CoV-2 an unprecedented threat to the human health worldwide. Recent clinal studies revealed a challenging situation of the SARS-CoV-2 pandemic. Although the magnitude of neutralizing antibody (NAb) responses correlated with COVID-19 severity, the NAb titers including IgM and IgA were subjected to rapid decline in most discharged COVID-19 patients, while virus might rebound in discharged patients who experienced low levels of prolonged viral replication in the gastrointestinal tract (Hu et al., 2020; Long et al., 2020). Moreover, SARS-CoV-2 is rapidly evolving with variants of increased infectivity and/or resistance to NAb, suggesting enormous challenges in the pandemic control (Korber et al., 2020; Pachetti et al., 2020; Weisblum et al., 2020). Hence, the combat against SARS-CoV-2 seems to require long-lasting efforts. In addition to the ongoing practice of physical distancing and investigative efforts on developing therapeutics and vaccines, precise medicine based on individual health condition may also help prevent risks of severe COVID-19.

Multiple clinical analyses have shown that there are high risks of severe COVID-19 in individuals with pre-existing comorbidities, such as obesity, diabetes and cardiovascular diseases (Bradley et al., 2020; Petrilli et al., 2020; Richardson et al., 2020). Notably, a growing number of evidences indicate that COVID-19 frequently involves gastrointestinal symptoms. 20–60% of COVID-19 patients presented gastrointestinal symptoms, which sometimes appeared even before the development of respiratory illness (Nobel et al., 2020; Pan et al., 2020; Redd et al., 2020). In a COVID-19 cohort in China, individuals with gastrointestinal symptoms were more likely to develop severe COVID-19 than those without gastrointestinal symptoms (Jin et al., 2020). Metabolic disorders and gastrointestinal illness are frequently associated with intestinal dysbiosis. Given the high frequency of gastrointestinal diseases in COVID-19 and the association between gastrointestinal symptoms and COVID-19 severity, it is possible that gut microenvironment can contribute to the alternative outcomes of COVID-19. A number of studies reported that SARS-CoV-2 could infect the gastrointestinal tract (Qian et al., 2020; Xiao et al., 2020), while the intestinal microecology and immune microenvironment may feedback to control the viral growth and virus-induced immunopathology in the pulmonary and cardiovascular systems, therefore, intestinal dysbiosis may contribute to regulating the severity of COVID-19 (Zuo et al., 2020c).

**FIGURE 1:**
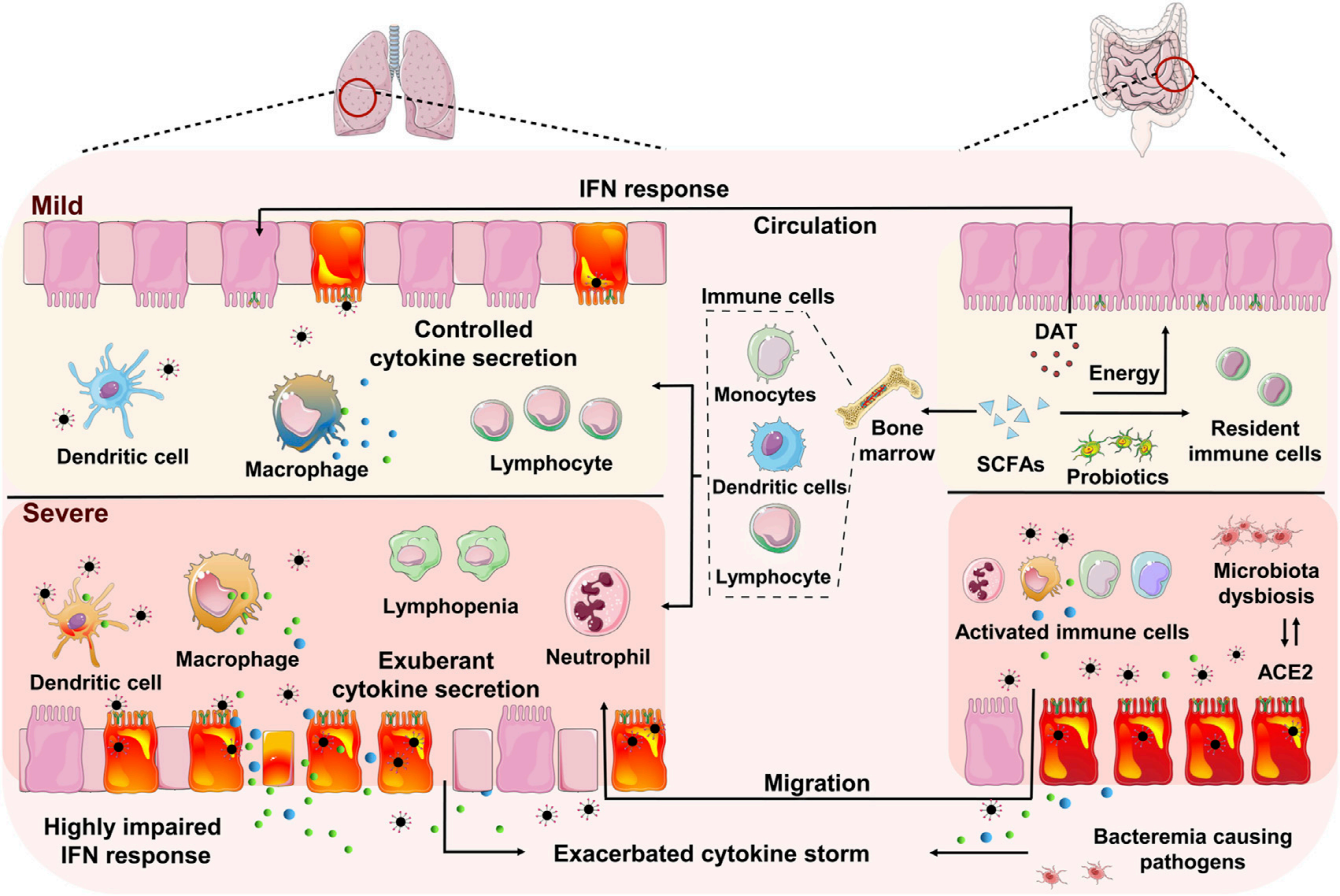
Schematic model of the gut-lung interaction in mild and severe COVID-19. The pulmonary immune responses in mild case were characterized by low numbers of infiltrating neutrophil and proinflammatory macrophages, along with a controlled level of proinflammatory cytokine secretion. While the predominant pulmonary immunopathology in severe COVID-19 is associated with significantly impaired interferon (IFN) responses, increased infiltration of neutrophils and proinflammatory macrophages, impaired antigen-presenting cells, reduction and functional exhaustion of cytotoxic T lymphocytes and exacerbated cytokine secretion. The intestine and lung communication enables trafficking of the immune cells and microbial metabolites along the gut-lung axis. The beneficial commensal bacteria and microbial metabolites, including short-chain fatty acids (SCFAs) and desaminotyrosine (DAT), may promote the antiviral innate immune responses and alleviate the immunopathogenic activities. Whereas the disorders of intestinal microecology may contribute to the pulmonary disease deterioration via bacteremia and the enrichment and spreading of the proinflammatory immune responses, in which the infected gut may be one of the origins of cytokine production.

According to the studies investigating the gut microbiota dysbiosis among many COVID-19 patients, as well as the “gut-lung axis” phenomena demonstrated in animal models infected by various respiratory virus, probiotics have been proposed as an alternative strategy to prevent SARS-CoV-2 infection and ameliorate COVID-19 symptoms (Wypych et al., 2019; Mak et al., 2020). In this review, we summarize the immune characteristics associated with severe COVID-19, the molecular evidence and characterization of enteric infection of SARS-CoV-2, and the association between the intestinal microenvironment and COVID-19 progression and prognosis (Figure 1). We focus to discuss the relationship between the severity of COVID-19 and the intestinal microenvironment as well as the potential of probiotics-based bacteriotherapy in preventing severe COVID-19.

## The Main Antiviral Responses in Mild/Moderate COVID-19 Patients

The majority of COVID-19 patients showed mild to moderate symptoms, e.g., fever, cough, fatigue and shortness of breath. Some of the infected people with pneumonia suddenly deteriorated into severe and even fatal respiratory conditions (Aggarwal et al., 2020; Huang et al., 2020). Reduced innate antiviral defenses coupled with exuberant inflammatory cytokine production were observed to be the main features of COVID-19 (Blanco-Melo et al., 2020; Sa Ribero et al., 2020).

### The Interferon Responses in Mild/Moderate COVID-19

Interferon (IFN) response to defend SARS-CoV-2 invasion was reported to be impaired with few production of IFNs while elevated expression of IFN-stimulated genes (ISGs), leading to the deficient control of early viral replication but promotion of the inflammatory responses (Blanco-Melo et al., 2020; Hadjadj et al., 2020; Sa Ribero et al., 2020). Upon infection of SARS-CoV-2 in a variety of respiratory cell lines and normal human bronchial epithelial (NHBE) cells, the viral load increased dramatically in a short time (Blanco-Melo et al., 2020). However, IFN-I and IFN-III were undetectable, while moderate level of ISGs was observed. Exogenous addition of INFβ to NHBE cells resulted in an increase in IFN-I response and a significant reduction in viral replication (Blanco-Melo et al., 2020). These results from *in vitro* cell culture assays and *ex vivo* infection of primary cells indicate that SARS-CoV-2 replication is sensitive to IFN-I, whereas there may be a virus-mediated IFN antagonism to attenuate antiviral response by host cells. Despite the lack of IFN expression, those respiratory cell lines and NHBE cells showed strong chemotactic and inflammatory responses to SARS-coV-2 infection. Consistently, COVID-19 patients exhibited low levels of IFN-I and -III and high chemokine signatures (Blanco-Melo et al., 2020). Compared to the biopsied lung tissue from healthy individuals, the transcriptional profiles of lung samples from COVID-19 patients (males older than 60°years) exhibited upregulation of a subset of ISGs with neglectable levels of IFN-I or IFN-III RNA expression. Metatranscriptomic sequencing with bronchoalveolar lavage fluid (BALF) cells from COVID-19 patients, community-acquired pneumonia (CAP) patients and healthy individuals also demonstrated a markedly elevated expression of ISGs in SARS-CoV-2 infected BALF cells than those of CAP patients, although the ISGs expression levels seemed to decrease over time (Zhou Z. et al., 2020). Among the elevated ISGs, despite the presence of the antiviral ISGs (*IFIT* and *IFITM* genes), proinflammatory ISGs were uniquely and predominantly elevated in SARS-CoV-2 infected patients, as compared to CAP patients. Although a robust induction of ISGs at mRNA was observed, the protein abundance of IFNs in BALF cells and sera of COVID-19 patients were quite subtle, below the levels of detection sensitivities of conventional ELISA (Blanco-Melo et al., 2020; Zhou Z. et al., 2020). However, using a highly sensitive ELISA assay, a significant increase in the plasma IFN-α level was observed in COVID-19 patients, which peaked 8°days after the onset of symptoms and regressed to baseline levels by day 20 (Arunachalam et al., 2020). In CITE-seq and bulk transcriptomic analyses of PBMCs, a strong correlation between the peripheral IFN-α concentration and the ISG responses in PBMCs over time was observed in COVID-19 patients, indicating that SARS-CoV-2 infection induces an early release of IFN-α from the lung followed by a moderate and transient wave of ISG expression in the lung and periphery (Arunachalam et al., 2020). Given that high SARS-CoV-2 loads were detected very early post symptom onsets, the discrepancy of upregulated expression of ISGs while nondetectable IFNs indicate that, SARS-CoV-2 may have developed countermeasures against the IFN system (Arunachalam et al., 2020; Zou et al., 2020).

### The Pulmonary and Peripheral Immune Profiles in Mild/Moderate COVID-19

In order to unveil the dysregulated immune profile resulting from SARS-CoV-2 invasion, the changes of pulmonary and peripheral immune cells in COVID-19 patients were investigated intensively (Liao et al., 2020; Zhou Z. et al., 2020). In mild and moderate COVID-19 patients, CD8^+^ T cells in the BALF were highly expanded and functionally competent with a tissue-resident memory T cell gene signature, while minimal infiltration of inflammatory monocytes into the BALF was observed (Liao et al., 2020). Moreover, a more pronounced recruitment of neutrophils into the lung was induced by SARS-CoV-2 in COVID-19 patients than those of CAP cases and healthy controls, according to the metatranscriptomic data of BALF cells (Zhou Z. et al., 2020).

Single-cell transcriptome analysis of PBMCs were performed to identify the changes of the circulating immune cells in COVID-19 patients, and the SARS-CoV-2 invasion elicited increased proportions of activated T cells, proliferative T cells and plasma B cells, and decreased ratios of naïve T cells, memory B cells and monocyte-derived dendritic cell (DC) subsets in the blood (Zhang J. Y. et al., 2020). Furthermore, detailed analyses were conducted by comparing the PBMCs from COVID-19 patients and those from age- and sex-matched healthy donors in two independent cohorts from Hong Kong and Atlanta (Arunachalam et al., 2020). These studies utilized phospho-CyTOF panel tests to characterize the cell phenotypes, and found that the frequencies of peripheral plasmablast and effector CD8^+^ T cells were significantly increased in all COVID-19 patients. Notably, the cytotoxic effector T cell subsets in the blood were found more significantly expanded in moderate COVID-19 patients compared to those from severe COVID-19 cases. Meanwhile, there was a significant reduction in the ratio of peripheral plasmacytoid DCs (pDC) in PBMCs of COVID-19 patients than those of healthy individuals, and the peripheral pDCs of COVID-19 individuals showed impaired IFN-producing capacity. The level of expression of phosphorylated-ribosomal protein S6 (pS6), a classic target of mTOR activation, was decreased in pDCs, which may in part explain their impaired capacity of producing IFN-α, since mTOR signaling is critical for IFN-α production in pDCs downstream of Toll-like receptor (TLR) stimulation (Cao et al., 2008; Arunachalam et al., 2020). In *ex vivo* cell culture experiments, it was determined that the peripheral pDCs from COVID-19 patients were functionally impaired in IFN-α and TNF-α production in response to a synthetic mixture of viral TLR ligands, in comparison to those from healthy individuals. Furthermore, genes involved in antigen presentation were significantly downregulated in blood myeloid cells during COVID-19. The levels of expression of HLA-DR and CD86 on monocytes and mDCs were remarkably decreased in COVID-19 patients, which were more pronounced in severe patients (Arunachalam et al., 2020).

### The Cytokine Responses in Mild/Moderate COVID-19

The immune dysfunction caused by SARS-CoV-2 was accompanied by local or global inflammatory cytokine storms (Mehta et al., 2020). Metatranscriptomic sequencing using BALF cells from 8 COVID-19 patients, 146 CAP patients and 20 healthy controls, revealed that the levels of expression of key ISGs, proinflammatory genes and chemokines were significantly elevated, suggesting that SARS-CoV-2 infection may cause hypercytokinemia in the lung (Zhou Z. et al., 2020). Chemokines were predominant among these upregulated cytokine-related genes in response to SARS-CoV-2 infection, including neutrophil recruiting chemokines (CXCL8, CXCL1, CXCL2), monocyte chemoattractant (CCL2, CCL7), CXCL17 and others, corresponding to the increased numbers of lung-infiltrating neutrophils and monocytes in COVID-19 patients. In COVID-19 patients, ultra-high viral loads were associated with the most pronounced upregulation of chemokine production. Besides chemokines, interleukin genes, such as *IL1RN* and *IL1B*, were also significantly upregulated in COVID-19 patients (Zhou Z. et al., 2020). With serum samples from healthy and SARS-CoV-2 infected individuals, it was further confirmed that SARS-CoV-2 could elicit a significant increase of proinflammatory cytokines, e.g., IL-6, IL-1Rα, as well as lymphocyte chemo-attractants, including CXCL9 and CXCL16 (recruit T or natural killer cells, respectively), CCL8 and CCL2 (attract monocytes and/or macrophages), and CXCL8 (an archetypal neutrophil chemoattractant), which collectively suggested that SARS-CoV-2 infection could cause hypercytokinemia (Zhou Z. et al., 2020).

Notably, despite the elevated level of circulating cytokines, substantial expression of proinflammatory cytokine genes was not detected in the peripheral monocytes, T cells or NK cells by single cell transcriptomic assay (Wilk et al., 2020), and impaired cytokine production by COVID-19 patient-derived peripheral myeloid cells and CD14^+^ monocytes in response to a viral TLR cocktail were confirmed in *ex vivo* cell culture experiments, regardless of the severity of COVID-19 donors (Arunachalam et al., 2020). These data suggest that the circulating cytokines may have a tissue origin, which is probably the inflamed lung. Notably, it has been observed that the expression levels of cytokine-related genes in moderate COVID-19 patients decreased over time (Zhou Z. et al., 2020). Significant upregulation of IL1RN and SOCS3, both of which encode cytokine signaling antagonists and contribute to the negative feedback loops, were observed in the BALF cells from COVID-19 patients (*n* = 8) (Zhou Z. et al., 2020). In an *in vivo* longitudinal study in ferrets, the SARS-CoV-2 infection responses were monitored over time by nasal wash for collection of the upper respiratory cells (Blanco-Melo et al., 2020). The cytokine response to SARS-CoV-2 infection was found to increase by 3°days post infection, and eventually decline to the baseline level by 14°days post infection, with the exception of IL-6 and IL-1Ra with sustained upregulation (Blanco-Melo et al., 2020). These observations suggest that the exuberant cytokine secretion due to SARS-CoV-2 infection may progressively diminish in mild/moderate COVID-19, or persist and contribute to detrimental outcomes in severe cases.

## The Immunopathogenic Responses Associated With Severe COVID-19 Patients

Compared with moderate COVID-19 patients, severe COVID-19 patients frequently had hypoalbuminemia and relatively high levels of alanine aminotransferase (ALT), aspartate aminotransferase (AST), lactate dehydrogenase (LDH), high-sensitivity C-reactive protein (hsCRP), ferritin, D-dimer and procalcitonin in the blood, suggestive of a significantly increased systemic inflammation (Chen G. et al., 2020). The hyperinflammatory response induced by SARS-CoV-2 in severe cases was consistently associated with highly impaired IFN response, substantial infiltration of proinflammatory monocytes into the lung, profound lymphopenia and persistent hypercytokinemica (Hadjadj et al., 2020; Mehta et al., 2020; Xu et al., 2020).

### The Dysregulated Immune Responses in the Lung and Periphery in Severe COVID-19

In order to better understand the mechanisms of SARS-CoV-2 infection and develop more effective means to control its associated mortality, a number of studies analyzed and compared the immune microenvironment of the lung and its periphery among moderate and severe COVID-19 patients (Chua et al., 2020; Xiong et al., 2020). In comparison to moderate cases, severe patients had higher proportions of macrophages and neutrophils, and lower ratios of mDCs, pDCs and T cells in their BALFs (Liao et al., 2020). Macrophages in BALFs from moderate COVID-19 patients produced relatively more T cell chemo-attractants, while those in the lungs of severe patients were relatively more proinflammatory with higher expression levels of cytokine and chemokine genes, which may contribute to the local inflammation and recruitment of more inflammatory monocytes and neutrophils that exacerbate immunopathology (Chua et al., 2020; Liao et al., 2020). High levels of proinflammatory cytokines, particularly IL-6, IL-8 and IL-1β, were found in the BALFs of severe COVID-19 patients. The subsets heterogeneity of pulmonary macrophages from moderate and critical COVID-19 patients was deeply analyzed and compared, it was found that the lung of severely infected individuals were enriched with macrophage subsets of peripheral monocyte-derived macrophages as well as macrophages with immunoregulatory and profibrotic functionality, indicating that the infiltrated and proinflammatory macrophages in the lung promote the acute inflammation and fibrotic complications in severely infected patients (Liao et al., 2020). In the lung of severe patients, there were lower fraction of CD8^+^ T cells and higher proportion of proliferating T cells within six major clusters of T and NK lymphocytes, than those from moderate COVID-19. Moreover, CD8^+^ T cells in BALFs from severely infected patients were less expanded than those in BALFs from moderate COVID-19 cases, indicating less cytotoxic T cell responses towards SARS-CoV-2 in severe COVID-19 than that with moderate infection (Liao et al., 2020).

The peripheral immune environment among moderate and severe COVID-19 patients were compared, and the immune dysregulation in the periphery of most severe COVID-19 cases was characterized by profound lymphopenia, impaired functionalities of antigen-presenting cells and cytotoxic immune cells, along with hypercytokinemia and hyperinflammation (Giamarellos-Bourboulis et al., 2020; Wilk et al., 2020; Zhang J. Y. et al., 2020). Acute SARS-CoV-2 infection triggered a broad reduction of circulating immune cells, including T cells, NK cells, monocytes and DCs (Chen G. et al., 2020; Zhou Z. et al., 2020). Particularly, the relative abundance of naïve T cells, mucosal-associated invariant T (MAIT) cells and monocyte-derived DCs decreased with the severity of COVID-19, whereas the proportions of proliferative T cells, plasma B cells, CD14^+^ monocytes and platelets increased with the disease severity (Zhang J. Y. et al., 2020). T cells showed higher cytotoxicity and more robust expansion in moderate patients, whereas higher exhaustion levels and less clonal expansion of T cells were seen in severe patients, and CD8^+^ T cells from severe patients were highly exhausted and functionally impaired. The reduction and functional exhaustion of T cells was closely related to the suppression of antigen-presentation cells, and the reduction in the expression levels of CD86 and HLA-DR on monocytes and mDCs was most pronounced in severe COVID-19 patients, evidenced by CITE-seq analysis, phspho-CyTOF and single-cell transcriptome data (Arunachalam et al., 2020). The effects of off-label Tocilizumab treatment of COVID-19 patients suggested that IL-6 plays a critical role in impairing antigen presentation and antiviral cytotoxicity in severe patients (Ohno et al., 2016; Diao et al., 2020; Mazzoni et al., 2020). Although neutralizing antibodies against SARS-CoV-2 were rapidly and abundantly generated in severe COVID-19 patients, the receptor binding domain (RBD)- and nucleocapsid protein (NP)- specific T cell responses were delayed at the acute stage, which may contribute to an uncontrolled virus spread and pathogenic immune responses (Altmann and Boyton, 2020; Zhou R. et al., 2020).

### The Exuberant Cytokine Storm Associated With Severe COVID-19

Severe COVID-19 patients were frequently associated with cytokine storm syndrome, which significantly contributed to the alveolar injury, multiple organ damage and even fatal outcomes (Zhou Y. et al., 2020). Many proinflammation cytokines, including IL-2, IL-6, IL-7, IL-10, granulocyte colony stimulating factor (GCSF) and TNF-α, were significantly increased in the periphery of severe COVID-19 patients (Chen N. et al., 2020; Huang et al., 2020; Liu J. et al., 2020). The concentration of circulating IL-6 was closely associated with the severity of COVID-19 symptoms, the impairment of immune cell cytotoxicity and the global T cell lymphopenia (Chen et al., 2020a; Mazzoni et al., 2020). In addition to IL-6, the elevation of three other proinflammatory and pulmonary injury-associated proteins, TNFSF14, EN-RAGE and oncostatin-M, were found in strong positive correlation with the severity of COVID-19 (Arunachalam et al., 2020). The proinflammatory cytokine storm could also trigger an increased coagulation via participating in endothelial dysfunction and leukocyte recruitment in the microvasculature, leading to the systematically impaired microcirculatory function in multiple organs of severe COVID-19 patients (Zhang Y. et al., 2020). Despite the elevated levels of circulating proinflammatory cytokines, the peripheral immune cells from COVID-19 patients were found to be impaired in their capacity to produce cytokines (Arunachalam et al., 2020). In multiple independent studies with CITE-seq and single-cell transcriptome assay, a lack of expression of type-I IFN and proinflammatory cytokine genes was repeatedly found in the PBMCs from severe COVID-19 patients, which is consistent with the functional data from *ex vivo* cell culture experiments (Arunachalam et al., 2020; Hadjadj et al., 2020; Wilk et al., 2020). These results suggest that the peripheral leukocytes and monocytes are not the major contributor to the cytokine storm in COVID-19, and these circulating cytokines are most likely released from the inflamed lung (Wilk et al., 2020).

The unique immune profiles in COVID-19 patients with an increase in proinflammatory mediators while decrease in innate immune responses in peripheral monocytes and DCs is similar to that of sepsis-like immune response (van der Poll et al., 2017). Accordingly, the serum samples of severe and intensive care unit (ICU) patients were detected with significantly higher levels of bacterial DNA and lipopolysaccharide (LPS), positively correlated with the levels of proinflammatory mediators, indicating that the exuberant cytokine storm may be further aggravated by the increased bacterial products from the lung or intestine.

## The Role of Enteric SARS-CoV-2 Infection in Severe COVID-19

### The Evidence of Intestinal Infection by SARS-CoV-2

**FIGURE 2:**
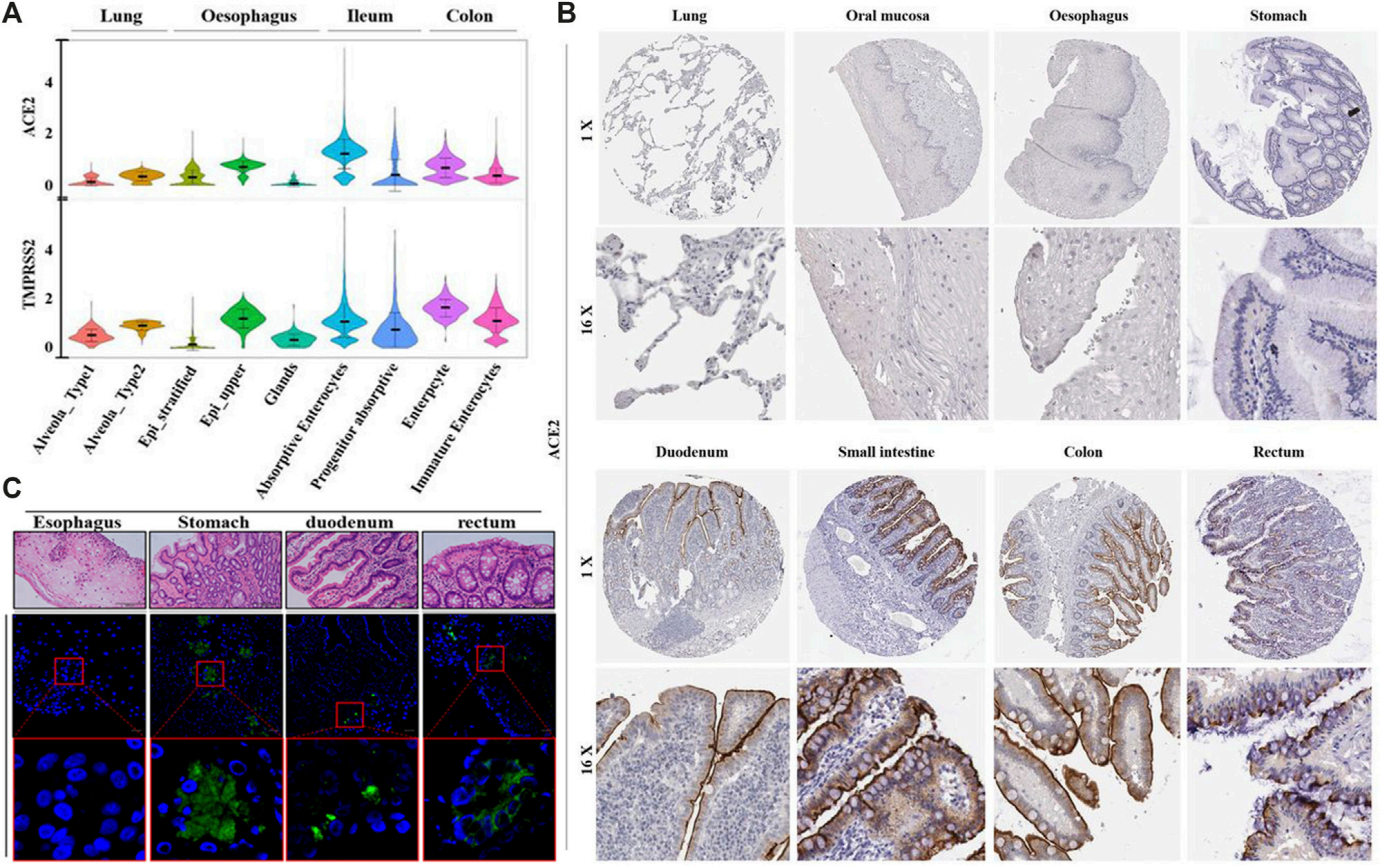
The abundance of ACE2, transmembrane serine protease (TMPRSS2) and tissue tropism of SARS-CoV-2 infection. **(A)** Violin plots for the levels of ACE2 and TMPRSS2 expression across various cell types among different tissues from healthy donors. **(B)** The distribution of ACE2 protein in the respiratory and digestive tracts from healthy individuals, by immunohistochemical imaging. **(C)** Images of gastrointestinal tissues from COVID-19 patients with immunofluorescent staining of SARS-CoV-2 NP. Reproduced with permission (Xiao et al., 2020; Zhang H. et al., 2020). Copyright 2020, the British Medical Journal and American Gastroenterological Association.

In addition to SARS-CoV-2 infection via the respiratory tract, it has been demonstrated that SARS-CoV-2 is capable of infecting the gastrointestinal tract, and the enteric infection could probably contribute to the disease progression of COVID-19 (Qian et al., 2020; Xiao et al., 2020). In order to better understand the tissue tropism of SARS-CoV-2 infection, multiple studies were conducted to identify the susceptible cell types with upregulated levels of virus entry genes across various tissues (Figure 2) (Du et al., 2020; Li et al., 2020; Zhang H. et al., 2020). The entry of SARS-CoV-2 into host cells relies on its binding on the host cell receptor and the subsequent activation of viral entry by a host protease (Hoffmann et al., 2020; Zhou P. et al., 2020). ACE2 was experimentally verified to be the cell receptor on the host cell membrane, via direct binding with the S protein of SARS-CoV-2 (Zhou P. et al., 2020). And the transmembrane serin protease 2 (TMPRSS2) was reported to be the main host cell protease, which cleaved the S protein of SARS-CoV-2 to initiate virus-cell membrane fusion to enable viral entry (Du et al., 2009). The mRNA and protein levels of ACE2 in bulk tissue from various human organs were examined, and ACE2 was found to be relatively more abundant in the lung and intestine (Hamming et al., 2004; Li et al., 2020; Wang et al., 2020). Single-cell transcriptomes of the lung and gastrointestinal samples of healthy people revealed that coexpression of ACE2 and TMPRSS2 were found not only in the lung alveolar type 2 (AT2) cells, esophagus upper epithelial and gland cells, but also in the absorptive enterocytes from the ileum and colon. Among all the ACE2-expressing cells in the digestive and respiratory tract, the expression of ACE2 was relatively higher in the ileum and colon (Zhang H. et al., 2020; Ziegler et al., 2020). These results indicate that the gastrointestinal tract is a potential route for SARS-CoV-2 infection, similar to the respiratory system.

Evidence for gastrointestinal infection by SARS-CoV-2 were provided by a series of *in vivo* and *in vitro* studies. Various gastrointestinal tissues were collected from a COVID-19 patient by endoscopy, and the presence of viral RNA was detected in the esophageal, gastric, duodenal and rectal specimens. ACE2 and viral nucleocapsid protein were also observed in the gastric, duodenal and rectal epithelia via immunofluorescent staining assay (Xiao et al., 2020). The enteric infection process of SARS-CoV-2 was further investigated in human intestinal organoids. The differentiated human enteroids and colonoids were observed to harbor abundant mRNA and protein levels of ACE2 and TMPRSS2, which sustained rapid replication of SARS-CoV-2, with dramatically increased viral load in the culture media over time (Lamers et al., 2020; Zhou J. et al., 2020). In a 2D monolayer culture system, ACE2 protein was observed to locate at the apical cell membrane of human enteroid. Consistently, SARS-CoV-2 were proved to preferentially infect the human intestinal epithelial cells (IECs) from the apical surface in comparison to the basolateral side, and were subsequently released from the apical side into the lumen, suggesting a potential viral shedding route along the digestive tract after enteric infection (Zang et al., 2020). Other than in the nasopharyngeal swabs and sputum, SARS-CoV-2 has been detected in the stool specimens of a substantial portion of COVID-19 patients (Du et al., 2020; Wang et al., 2020; Zhou J. et al., 2020). In a cohort of 73 COVID-19 patients from China, 53% of them had SARS-CoV-2 RNA in their fecal samples, and 23% of these patients remained SARS-CoV-2 positive in their stools even after the viral RNA was undetectable in their respiratory tract (Xiao et al., 2020). The duration of SARS-CoV-2 positivity by RT-PCR in the stool specimens was reported to be significantly longer than that of respiratory and serum samples, among a cohort of 96 COVID-19 patients (Zheng et al., 2020). These results provide evidence for gastrointestinal infection of SARS-CoV-2, suggesting a considerable contribution of SARS-CoV-2 enteric infection to the overall disease progression.

### The Correlation Between Enteric SARS-CoV-2 Infection and COVID-19 Severity

The biased distribution and multifunctional role of ACE2 links the intestinal microenvironment to the disease progression of COVID-19. Both ACE2 expression and COVID-19 severity have been demonstrated with interaction with the intestinal flora. The gut microbiota was reported to influence the expression level of colonic ACE2 (Yang et al., 2020). *Coprobacillus*, which was positively correlated to the severity of COVID-19, was shown to upregulate the expression of ACE2 in the murine intestine (Geva-Zatorsky et al., 2017). The fecal virus load of SARS-CoV-2 in severe COVID-19 patients were found to be inversely correlated with 4 *Bacteroides* species, *i.e.*, *Bacteroides dorei*, *Bacteroides thetaiotaomicron*, *Bacteroides massiliensis* and *Bacteroides ovatus*, which could downregulate the expression of ACE2 in the murine colon (Geva-Zatorsky et al., 2017; Zuo et al., 2020c). Hence, the gut microbiome of individuals with higher risk of severe COVID-19 were supposed to promote enteric infection by SARS-CoV-2 through stimulating higher ACE2 exposure in the digestive tract. In addition to the receptor for spike protein of SARS-CoV-2, ACE2 can function as a chaperone for the neutral amino acid transporter B^0^AT1 on enterocytes and regulate the microbial homeostasis. One potential cause to the dysregulated intestinal microbiota under severe COVID-19 could be the substantial internalization of ACE2 receptor during ACE2-mediated viral entry into the host cells. Without the membrane ACE2, the decreased absorption of tryptophan by B^0^AT1 triggered a reduction in the antimicrobial peptides secretion via mTOR signaling, resulting in the gut microbiota dysbiosis and higher susceptibility to intestinal inflammation (Hashimoto et al., 2012).

**FIGURE 3:**
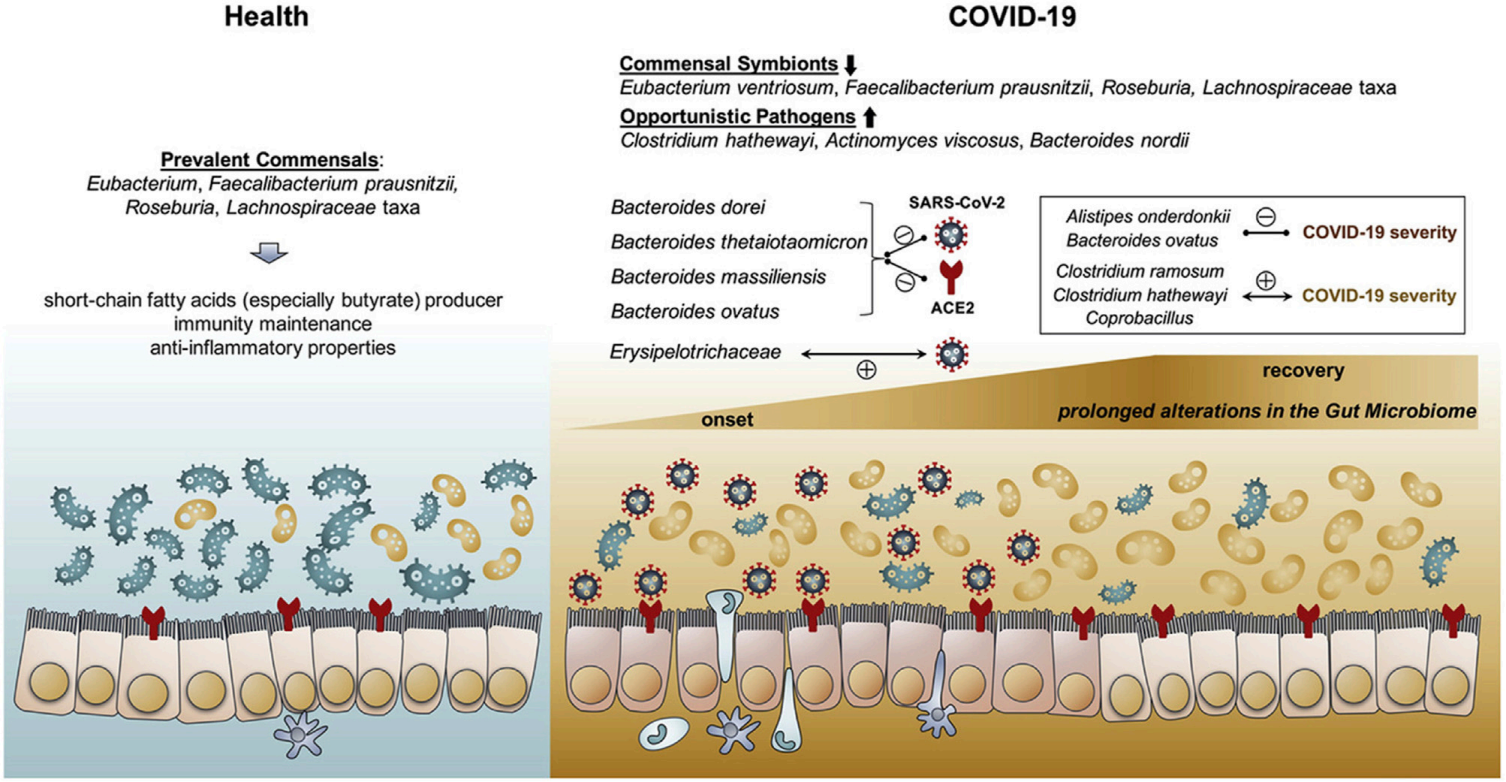
Schematic summary of the gut microbiome alterations in COVID-19. A. The gut flora in healthy people are enriched with short-chain fatty acids (SCFAs) producing bacteria. While COVID-19 patients are frequently accompanied with prolonged gut microbial dysbiosis during and even after the disease course, with elevated abundance of opportunistic pathogens and decreased abundance of beneficial commensals. Reproduced with permission (Zuo et al., 2020c). Copyright 2020, American Gastroenterological Association.

The intestinal microbiota of COVID-19 patients was reported to be significantly altered by SARS-CoV-2 infection (Gu et al., 2020; Zuo et al., 2020b; Zuo et al., 2020c). In comparison to that of healthy controls, the gut microbiomes of COVID-19 patients were generally characterized by reduced microbial flora diversity, enriched opportunistic pathogens and depleted beneficial commensals (Figure 3), by means of shotgun metagenomics or 16S rRNA sequencing analysis of fecal specimens (Gu et al., 2020; Zuo et al., 2020a; Zuo et al., 2020b; Zuo et al., 2020c). In order to investigate the correlation between intestinal microbiota dysbiosis and COVID-19 severity, a cohort of COVID-19 patients with various severity were monitored for their fecal microbiomes during hospitalization (Zuo et al., 2020c). COVID-19 patients were enriched with opportunistic pathogens which were known to cause secondary bacterial infection and bacteremia. Through assessing the baseline fecal microbiome (from the first stool sample collected after hospitalization) of 7 COVID-19 cases without antibiotics use, the gut microbes with the strongest positive correlation with disease severity were *Coprobacillus* bacteria, *Clostridium ramosum* and *Clostridium hathewayi*, and the latter two species have been reported to elicit bacteremia (Elsayed and Zhang, 2004; Leal et al., 2008). These results are consistent with the previous findings that significantly higher levels of bacterial DNA and LPS appeared in the plasma of severe COVID-19 patients, and the baseline abundance of bacteremia-causing pathogens could likely contribute to the exacerbated cytokine storm and disease deterioration.

Robust viral replication in the gastrointestinal tract and the subsequent virus shedding were found to be closely associated with gut dysbiosis among COVID-19 patients (Zuo et al., 2020a). In comparison to COVID-19 patients with low-to-none SARS-CoV-2 infectivity in their stools, the fecal specimens with high SARS-CoV-2 infectivity had lower abundance of short-chain fatty acids (SCFAs) producing bacteria (*Parabacteroides merdae*, *Bacteroides stercoris*, *Alistipes onderdonkii* and *Lachnospiraceae bacterium*) as well as higher abundance of opportunistic pathogens (*Collinsella aerofaciens*, *Collinsella tanakaei*, *Streptococcus infantis* and *Morganella morganii*), indicating an intimate connection between uncontrolled viral infection in the intestine with gut microflora dysbiosis (Zuo et al., 2020a). SCFAs are critical microbial metabolites which could shape the mucosal immune responses, and decreased levels of SCFAs are indicative of imbalanced and proinflammatory gut microbiota (Koh et al., 2016). In human intestinal organoids, the enterocytes infected by SARS-CoV-2 exhibited an elevated expression of cytokine genes and ISGs along with relatively low expression of type I and III IFNs, resembling the dysregulated immune signature of SARS-CoV-2 invaded lung (Lamers et al., 2020). In *ex vivo* human intestinal tissues, SARS-CoV-2 infection significantly upregulated the expression of proinflammatory mediators, including IL12, IL8, MCP-1, MIP1α, CXCL2, CXCL5, and CXCL9 (Chua et al., 2020). Consistently, intestinal edema with significant infiltration of plasma cells and lymphocytes were observed in the lamina propria of gastrointestinal mucosa in COVID-19 patients (Xiao et al., 2020). It is reasonable to speculate that the enteric infection by SARS-CoV-2 could trigger a proinflammatory response and hypercytokinemia in the intestine, and the inflamed gut is probably one of the origins of the circulating cytokines besides the lung.

## Perspectives of Bacteriotherapy in Preventing or Alleviating Severe COVID-19

According to the clinical statistical analysis of large COVID-19 cohorts, eldly people and individuals with metabolic comorbidities including obesity, diabetes, hypertension and cardiovascular diseases are at relatively higher risk of developing severe COVID-19, which are frequently accompanied by gut microbiota dysbiosis (Chen et al., 2020b; Liu H. et al., 2020; Zhao et al., 2020). A growing body of research has shown that the intestinal microbiota disorders significantly correlate with and promote the development of various metabolic diseases. For instance, intestinal dysbiosis in type 2 diabetes (T2D) was generally characterized by increased abundance of proinflammatory bacteria and decreased levels of beneficial commensal bacteria (Tilg et al., 2020). Similarly, the gastrointestinal microbiome of hypertension was frequently associated with enriched opportunistic pathogens as well as reduced beneficial SCFA-producing bacteria (Yan et al., 2017). The recent studies on the molecular mechanisms underlying the relationship between intestinal microenvironment and susceptibility to developing severe COVID-19 were mainly focused on the potential interaction among ACE2, intestinal microbiome and local proinflammatory responses, according to the few studies on the gastrointestinal specimens of COVID-19 patients. A plenty of studies on various respiratory viruses have proved that the pulmonary antiviral capacity is associated with the intestinal microbiota hemostasis, and the intestinal microecology have a long-reaching impact on the pulmonary antiviral capacity through the “gut-lung axis”, besides its role in shaping the mucosal immune responses in the intestine (Chiu et al., 2017). The gut microbiota could impact the physiological outcomes of viral infection at multiple levels and stages, via modulating the expression levels the IFN signaling components and cell-intrinsic antiviral effects of the lung epithelia during the early control of viral replication, and through influencing the innate and adaptive immune reactions and immunopathology later on (Mjosberg and Rao, 2018; Wypych et al., 2019). Hence, the disorders of the gut microecology probably play a significant role in impacting the early control of SARS-CoV-2 and the associated immunopathogenic responses, and consequently influencing the disease progression of COVID-19.

Regeneration of gut microbiota hemostasis in individuals with chronic metabolic syndromes could possibly decrease their risk of getting severe COVID-19. Moreover, precise treatment of the gut flora dysbiosis in COVID-19 patients may contribute to prevent the disease deterioration. Antibiotics were frequently used to prevent or treat the secondary bacterial infection after SARS-CoV-2 infection. However, the gut microbiome disorders of severe COVID-19 patients were worsened by antibiotics use, with a flora configuration deviated farther away from that of the healthy microbiome and a further depletion of multiple beneficial symbionts (Zuo et al., 2020c). Antibiotics-treated mice infected by respiratory viruses generally exhibited severer lung damage and higher mortality, with impaired innate and adaptive immune responses for viral clearance. Obviously, the antibiotics without specificity in bacteria species should be cautiously applied. In a cohort of COVID-19 patients treated with hydroxychloroquine, antibiotics and tocilizumab, alone or in combination, the group with additional oral intake of probiotics (*Streptococcus thermophilus*, *Lactobacilli acidophilus*, *L. helveticus*, *L. paracasei*, *L. plantarum*, *L. brevis*, *Bifidobacteria lactis*) had a significantly reduced risk of developing respiratory failure by around 87.5%, in comparison to the group without additional bacteriotherapy (d'Ettorre et al., 2020). Whereas a previous study exhibited nonsignificant effects of probiotics in promoting gut microbiota hemostasis. In a randomized, placebo-controlled trial with a large number of participants, a 21-days treatment with *Lactobacilli* and *Bifidobacteria* did not reduce antibiotic-associated diarrhea (Allen et al., 2013). Although probiotics treatment has been proposed as a potential therapy to prevent COVID-19 deterioration, the rationale for designing probiotics formulation with more targeted efficacy to attenuating COVID-19 progression awaits further investigations (Mak et al., 2020).

Regarding the uniquely dysregulated immune profiles and the intestinal microenvironment associated with severe COVID-19, a more targeted and time-specific probiotics formulation is desirable to prevent or alleviate severe outcomes of the infection. Preceding or at the onset of SARS-CoV-2 infection, a probiotics formulation capable of promoting gut flora homeostasis as well as elevating the innate antiviral capacity of the lung will be of potential help contributing to early control of SARS-CoV-2 replication. The gut microbiota was reported to impact the expression of lung stromal IFN signaling components preceding the viral infection, and hence influence the cell-intrinsic antiviral effects of the lung epithelia at the steady state (Bradley et al., 2019). A human commensal bacteria *Clostridium orbiscindens* exerted a protective impact of defending against pulmonary viruses through its metabolite desaminotyrosine (DAT) (Steed et al., 2017). It was found that DAT had protective effects against respiratory viral infection at the early stage, through priming the pulmonary type I IFN responses, while it could lead to a worse outcome at the later stage (Steed et al., 2017). At the later stage of COVID-19, it may be beneficial for these patients to receive probiotics cocktails with functions of downregulating the colonic ACE2 expression, attenuating the immunopathology and reducing the bacteremia-inducing pathogens. Four *Bacteroides* species, *i.e.*, *Bacteroides dorei*, *Bacteroides thetaiotaomicron*, *Bacteroides massiliensis* and *Bacteroides ovatus* were reported to downregulate ACE2 expression in the murine colon, and were found to be inversely correlated with the fecal SARS-CoV-2 loads in severe COVID-19 patients (Geva-Zatorsky et al., 2017; Zuo et al., 2020c). Besides probiotics, prebiotics are optional choice for enriching SCFAs producing beneficial bacteria, which was demonstrated to improve the survival of influenza-infected mice by suppressing neutrophil infiltration into the lung and enhancing CD8^+^ T cell effector function (Trompette et al., 2018; Infusino et al., 2020). To eliminate the bacteremia-inducing pathogens, phages and antimicrobial peptides with much higher specificity than antibiotics are alternative powerful tools (Kaabi and Musafer, 2019; Xiong et al., 2019).
